# Conceptual design of the superconducting magnet for the 250 MeV proton cyclotron

**DOI:** 10.1186/s40064-016-2340-0

**Published:** 2016-05-23

**Authors:** Yong Ren, Xiaogang Liu, Xiang Gao

**Affiliations:** Institute of Plasma Physics, Hefei Institutes of Physical Science, Chinese Academy of Sciences, PO Box 1126, Hefei, 230031 Anhui People’s Republic of China

**Keywords:** Cryostat, HTS current lead, Proton cyclotron, Superconducting magnet

## Abstract

**Background:**

The superconducting cyclotron is of great importance to treat cancer parts of the body. To reduce the operation costs, a superconducting magnet system for the 250 MeV proton cyclotron was designed to confirm the feasibility of the superconducting cyclotron.

**Findings:**

The superconducting magnet system consists of a pair of split coils, the cryostat and a pair of binary high temperature superconductor current leads. The superconducting magnet can reach a central magnetic field of about 1.155 T at 160 A. The three GM cryocooler with cooling capacities of 1.5 W at 4.5 K and 35 W at 50 K and one GM cryocooler of 100 W at 50 K were adopted to cool the superconducting magnet system through the thermosiphon technology.

**Conclusion:**

The four GM cryocoolers were used to cool the superconducting magnet to realize zero evaporation of the liquid helium.

## Background

Cancer is one of the main causes of death in the world today (Siegel et al. 2014). In the field of cancer treatment, proton beams often offer an improved dose distribution compared with the commonly used photon and electron beams, and thus enable dose escalation while sparing normal tissues (Schulz-Ertner and Tsujii 2007; Ma 2009). The proton beam therapy has the unique merits mentioned above that makes it particularly attractive for the treatment of pediatric cancers, cancers in the eye, cancer of skull base, and spine cancer (Efstathiou et al. 2013; Levin et al. 2005). The 250 MeV superconducting cyclotron for proton therapy is being designed due to the advantages with high magnetic field, low operation costs and more compactness compared with the conventional magnet technology (Kang et al. 2010; Newhauser and Zhang 2015; Choi et al. 2010). The design goals of the superconducting cyclotron include high reliability, low activation, easy maintenance and easy to use. To confirm the feasibility of the superconducting cyclotron, a superconducting magnet for the 250 MeV cyclotron is being designed to evaluate the electromagnetic and cryogenic properties. The superconducting magnet system consists of a pair of split coils, cryostat, a pair of HTS current leads, and four GM cryocoolers. The cryogenic system needs to be designed to realize zero evaporation of the liquid helium.

In this paper, the design of the superconducting magnet system is described. Also, the electromagnetic and thermal performance of the superconducting magnet and the thermal characteristics of the cryogenic system are analyzed.

## Description of the superconducting coils

The proton therapy is of great interest due to its superior spatial dose distribution to a tumor (Newhauser and Zhang 2015). The proton beam can be used as a tool to treat the eye cancer, lung cancer, and head cancer etc. The proton energy depends on where the cancers are located. The generally accepted treatment region is greater than 300 mm in depth. The proton beam must have enough energy to treat cancer parts of the body. The proton beam energy of 250 MeV can be used to treat in theory the tissues of 400 mm. The purpose of this work for selecting the proton energy of 250 MeV was to consider the peak dose located at an approximate 400 mm depth for medical application. To obtain the energy of 250 MeV for the proton beam, the speed of the proton is accelerated to 60 % speed of light with a dedicated accelerator. By including the relativistic effect, the final orbit radius of the accelerated proton beam can be expressed as (1),

1 $$ r = \frac{{\sqrt {E \cdot (E + 2E_{0} )} }}{300 \cdot Z \cdot B} $$

where *E*_0_ and *E* are the rest energy and kinetic energy of the proton, *Z* is the atomic number of the proton and *B* is the average magnetic field intensity at the radius r.

**Fig. 1:**
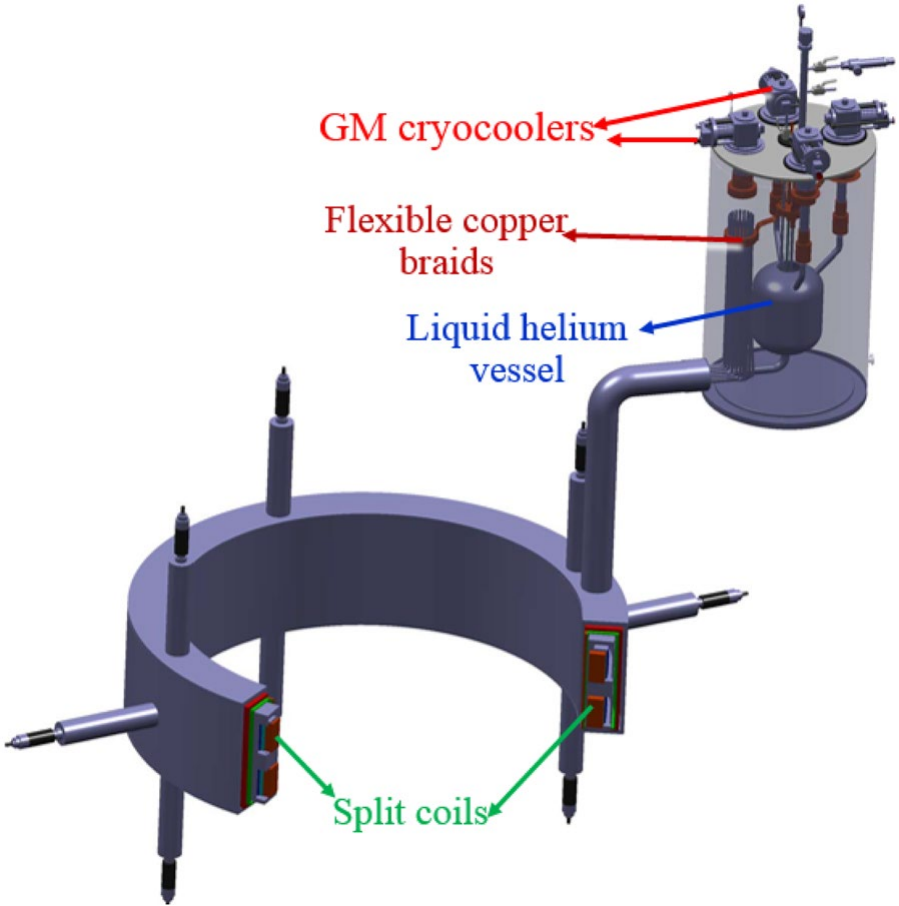
Superconducting magnet system for the 250 MeV proton accelerator

**Fig. 2:**
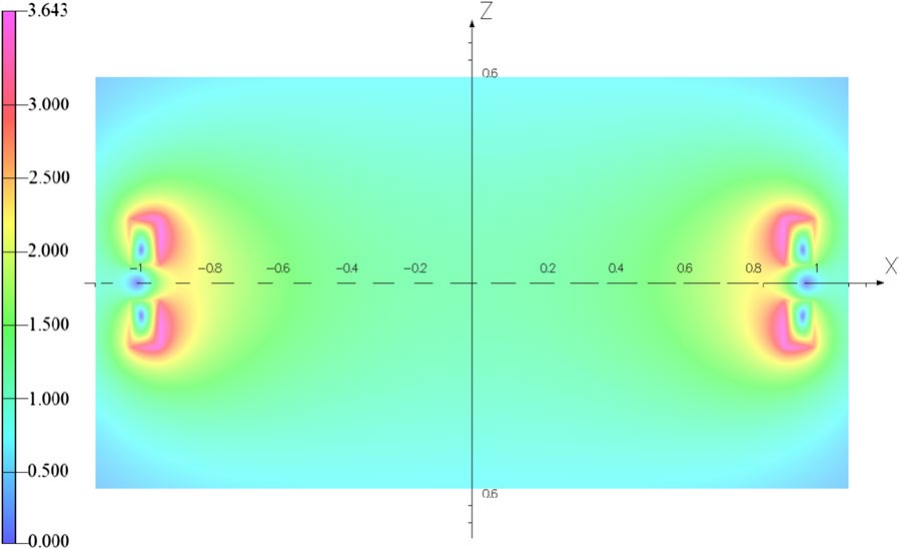
Magnetic field distribution of the superconducting magnet without iron yoke in the cross sectional plane (unit: T)

**Fig. 3:**
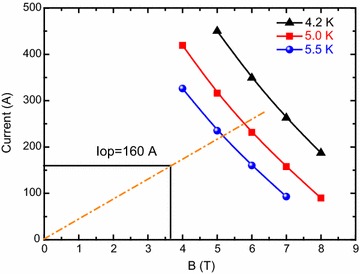
Load lines of the superconducting magnet (“Iop” stands for the operating current)

For the proton, the *E*_0_ and Z are about 938 MeV and 1. Therefore, the magnetic field at radii of 0.81 m is about 3 T for the proton cyclotron. To reduce the power consumption, the superconducting magnet was adopted. To confirm the feasibility of the superconducting cyclotron, a superconducting magnet is being designed to evaluate the electromagnetic and thermal characteristics. The superconducting magnet for the cyclotron is composed of a pair of split coils made of NbTi superconductors. Figure 1 shows the superconducting magnet system for the 250 MeV proton accelerator. The magnet can generate a central magnetic field of 1.155 T at 160 A. The maximum magnetic field of the superconducting magnet is about 3.643 T located at the inner surface of the first coil with a displacement of 149.4 mm from the mid-plane. The total inductance and stored magnetic energy of the magnet are about 344.2 H and 4.406 MJ at 160 A. Table 1 lists the design parameters of the split coils. Figure 2 shows the magnetic field distribution of the split coils without the iron in the cross sectional plane. The ratio of copper to superconductor (Cu/SC ratio) in the cross-section of the NbTi strand is about 7.0. Figure 3 shows the load line of the superconducting magnet. It is shown that there are sufficient temperature margins during the magnet operation. The minimum temperature margin of the superconducting magnet is above 1.5 K.

**Table 1:** Design parameters of the superconducting magnet

Coil	S1	S2
Strand	NbTi	NbTi
Strand dimension (mm)	1.80 × 1.20	1.80 × 1.20
Cu/SC ratio	7	7
Insulator	Formvar	
Inner radii (mm)	910	910
Outer radii (mm)	999.5	999.5
Mid-plane (mm)	−119.9	119.9
Height (mm)	136.8	136.8
Turns	5624	5624
Operating current (A)	160	
Inductance (H)	300	
Stored energy (MJ)	4.406	
Central field (T)	1.155

When energized, a large electromagnetic force and stress are generated in the superconducting magnet. To improve the mechanical stability of the superconducting magnet, a pretension of 80 MPa was exerted on the coils during winding (Chen et al. 2010).

## Cryostat design of the 250 MeV superconducting cyclotron

The cryogenic system of the superconducting magnet system is composed of the cryostat, GM cryocooler, coldbox, and magnet feeder. The GM cryocooler was used to cool the superconducting magnet system through thermosiphon technology. The three GM cryocoolers with cooling capacity of 1.5 W at 4.2 K and 35 W at 50 K are located at the top of the cooling cryostat to recondense the helium gas from the superconducting coil cryostat. The fourth GM cryocooler with 100 W at 50 K was used to cool the thermal shield and the other structure. The flexible copper braids were used to connect the cold head of the GM cryocoolers to the conduction structure in order to avoid any damage due to thermal contraction.

The thermosiphon tube can also be used as a relief channel during a magnet quench. To reduce the operational costs of the superconducting magnet, it is required to reduce the heat load from the radiation, residual gases and thermal conduction of the superconducting magnet system. The cryogenic system should be optimized to reduce the heat load:

1. To reduce the heat load from the residual gases, the vacuum pressure should be below 2 * 10^−4^ Pa.
2. In order to reduce the radiation heat load, an aluminum foil with a thickness of 20 μm has been applied to the surface of the coil case.
3. 40 Layers of superinsulation material is required to reduce the radiation heat.
4. The Al 1100 material of 6 mm in thickness with high thermal conductivity was selected as the thermal radiation shield for the cryostat. The maximum temperature of the thermal shield should not exceed 60 K. The six copper tubes as cooling channels were welded on the surface of the thermal shield.
5. The 12 tie rods with Carbon fireglass material were adopted to support the cold mass of the superconducting coils and to reduce the thermal conduction. The support structure for the cold mass is composed of eight longitudinal and four radial tie rods.
6. Pre-cooling the superconducting magnet can be accomplished through two different methods. In the first option, approximately 1000 L of liquid nitrogen is used to cool the magnet to 77 K. Subsequently a combination of nitrogen gas and helium gas is used to exhaust the liquid nitrogen. Finally, liquid helium is admitted into the cryostat. In the second option, only helium gas, cooled by the cryocooler is circulated through the superconducting magnet. The second option avoids the challenge of removing all the nitrogen, but it requires a longer time.

The calculated heat load from the thermal shield and support structure is about 30 W at 60 K. The heat load from the HTS current leads is below 0.3 W at 4.2 K and 25 W at 60 K, which will be described in the next section. The maximum heat load from proton beam losses is below 2.0 W at 4.2 K. The heat loads from the proton losses are not a real source in the present study, which can be simulated with the heater during the magnet operation. Therefore, there are sufficient cooling capacity with four GM cryocoolers of 4.5 W at 4.2 K and 205 W at 50 K to cool the superconducting magnet system to realize zero evaporation of the liquid helium.

## Design of a pair of binary HTS current leads

To reduce the heat leak from the room temperature to the liquid helium temperature, a pair of binary HTS current leads were adopted. The binary current leads consists of two parts, i.e., the warm end section and the cold end section. The OFHC copper material with RRR of about 50 was adopted for the warm end to connect the power supplies. To gain enough safety margin, the maximum allowable current density of the OFHC copper is limited to 10 A/mm^2^. The four Bi-2223/Ag–Au HTS tapes were adopted to reduce the heat load for the cold end. The stacks of Bi-2223/Ag–Au tapes need to be mechanically supported by stainless steel tube for the cold end section. The stainless steel can be used as a current shunt during a quench due to its larger heat capacity.

The warm end section of the HTS tapes can be cooled with the 1st stage cold head of the GM cryocoolers. The connected material between the warm end of the HTS tapes and the copper plate used for heat conduction of the 1st stage cold head of the GM cryocoolers is the In/AlN/In structure. The structure has two advantages, i.e., high thermal conductivity and excellent electrical insulation. The cold end of the HTS can be cooled with the 2nd stage of the GM cryocoolers or the liquid helium. For safety, the cold end of the HTS tapes can be cooled with liquid helium. Table 2 lists the design parameters of a pair of binary HTS current leads. The polyimide film was used to insulate the current leads to ground.

**Table 2:** Design parameters of a pair of HTS current leads

Material	HTS	OFHC copper
Maximum operating current (A)	250	250
Ground insulation (V)	500	500
HTS	4 × Bi-2223/Ag–Au	–
Joint resistance between HTS and NbTi/Cu (μΩ)	<0.2	<1
Operating temperature (K)	5~60	60~300
Heat load (W)	<0.3 @4.2 K	<25 @60 K

The temperature sensor of Pt was adopted to monitor the operating temperature of the HTS. The HTS should be operated at a temperature below 70 K. The quench protection system for the HTS element is activated if the operating temperature exceeds 90 K, or the threshold voltage of the HTS element exceeds 2 mV.

## Quench protection design of the 250 MeV superconducting cyclotron

The superconducting magnet is powered with a power supply. The stored energy of the superconducting magnet is about 4.406 MJ. To protect the superconducting magnet against damage during a quench, the appropriate quench protection is required. The quench protection circuit needs to be designed to limit the quench hot spot temperature and the quench voltage. The two split coils are subdivided into 4 sections to limit the quench voltage. Each section is in parallel with a back-to-back diode and a dump resistor. To accelerate the quench propagation, the quench heater was adopted. As a further study, we will describe the quench protection design and the relevant quench analysis in detail in the following paper.

## Conclusion

In this paper, the conceptual design of a superconducting magnet for the 250 MeV proton cyclotron has been described. The superconducting magnet consists of two split coils made of NbTi superconductor with a large stored energy of 3.84 MJ. The relevant performance analysis of the superconducting magnet system were described. The thermal analysis shows that the superconducting magnet can realize zero evaporation of the liquid helium. Design of the superconducting magnet system is in progress and will be fabricated in future.
